# Supplementary material for: Splice-Junction-Based Mapping of Alternative Isoforms in the Human Proteome
Source: Cell Rep. Author manuscript; Available in PMC 2020 Jan 15. (PMC6961840; doi:10.1016/j.celrep.2019.11.026)
Supplement: 3 [file NIHMS1546469-supplement-3.zip › DF2/PXD0010154/Ovary-159-Q9UIS9-REGASSPVQVPGPVAASTEALLQAVDPGLPSVK.pdf]

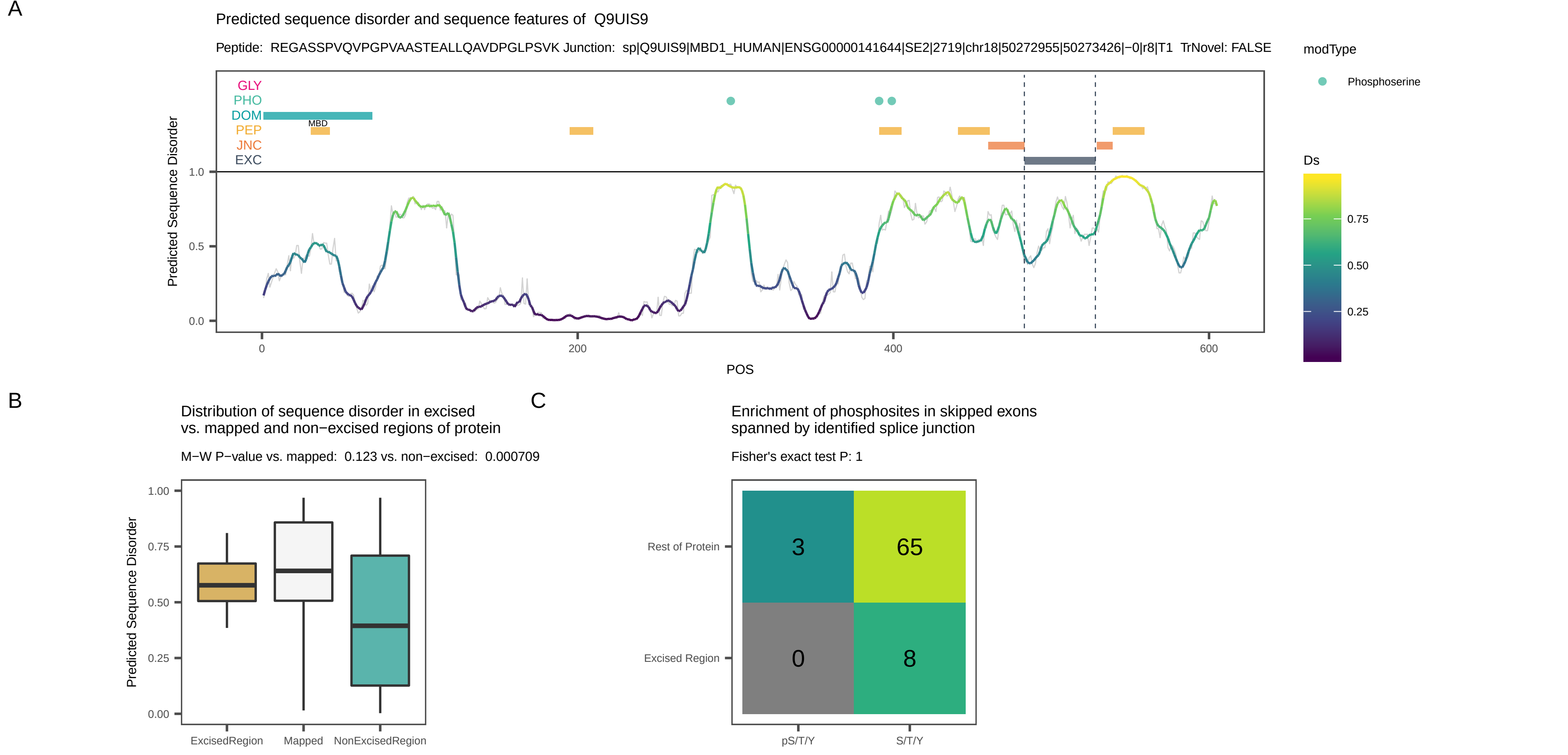
A
Predicted sequence disorder and sequence features of Q9UIS9
Peptide: REGASSPVQVPGPVAASTEALLQAVDPGLPSVK Junction: sp|Q9UIS9|MBD1_HUMAN|ENSG00000141644|SE2|2719|chr18|50272955|50273426|−0|r8|T1 TrNovel: FALSE
GLY
PHO
DOM
PEP
JNC
EXC
MBD
Predicted Sequence Disorder
1.0
0.5
0.0
0
200
400
600
POS
modType
Phosphoserine
Ds
0.75
0.50
0.25
B
Distribution of sequence disorder in excised vs. mapped and non−excised regions of protein
M−W P−value vs. mapped: 0.123 vs. non−excised: 0.000709
Predicted Sequence Disorder
1.00
0.75
0.50
0.25
0.00
ExcisedRegion
Mapped
NonExcisedRegion
C
Enrichment of phosphosites in skipped exons spanned by identified splice junction
Fisher's exact test P: 1
Rest of Protein
Excised Region
3
65
0
8
pS/T/Y
S/T/Y
